# Controversies about COVID-19 and anticancer treatment with immune checkpoint inhibitors

**DOI:** 10.2217/imt-2020-0067

**Published:** 2020-03-26

**Authors:** Melissa Bersanelli

**Affiliations:** 1 Medical Oncology Unit, University Hospital of Parma, Parma, Italy; 2 Medicine & Surgery Department, University of Parma, Parma, Italy

**Keywords:** anti-CTLA-4, anti-PD-1, anti-PD-L1, cancer patients, COVID-19, immune checkpoint inhibitors, immunotherapy, SARS-CoV-2, tocilizumab, viral infection

## Corona virus disease-19 pandemic & cancer patients

On 11 March, the WHO formally declared the corona virus disease-19 (COVID-19) outbreak a pandemic [1]. After the first cluster of cases emerged from Wuhan, in China, at the end of 2019, up today almost 287000 cases of infections from severe acute respiratory syndrome coronavirus 2 (SARS-CoV-2) have been diagnosed across all five continents in the last few months [2,3].

COVID-19 morbidity and mortality have been linked to elderly age and comorbidities, leading to a poorer outcome to the viral infection for frail patients and more often resulting in hospitalization, intensive care unit admittance and need for invasive tracheal intubation [4]. Among such individuals, cancer patients represent a large subgroup at high risk of developing coronavirus infection and its severe complications. A recent nationwide analysis in China demonstrated that, of 1590 COVID-19 cases from 575 hospitals, 18 had a history of cancer (1 vs 0.29% of cancer incidence in the overall Chinese population, respectively), with lung cancer as the most frequent diagnosis [5]. Patients with cancer were observed to have a higher risk of severe events compared with patients without cancer (39 vs 8%; p = 0.0003). Moreover, cancer patients who underwent recent chemotherapy or surgery had a higher risk of clinically severe events than did those not receiving treatment. With the limit of a small sample size, the authors concluded that patients with cancer might have a higher risk of COVID-19, and poorer outcomes, than individuals without cancer. As a consequence, they recommended to consider an intentional postponing of adjuvant chemotherapy or elective surgery for stable cancer in endemic areas [5].

Nevertheless, as subsequently highlighted by other authors, the true incidence of COVID-19 in patients with cancer would be more informative in assessing whether such patients have an increased risk (and morbidity) from this viral illness [6]. Furthermore, the limited cancer patient population described in this first report from the literature, was curiously characterized by the lack of individuals receiving anticancer immunotherapy. Indeed, only chemotherapy and surgery were cited among treatments received by patients in the month prior to developing COVID-19. Maybe, this could simply be due to the casualty of a small sample, or otherwise, it could suggest that cancer patients receiving immunotherapy are less prone to develop COVID-19 or to be admitted in hospital due to severe coronavirus symptoms. Currently, we are aware of the probably higher incidence of misdiagnosed coronavirus infections compared with that reported and updated every day; it is likely that a great portion of healthy and young population develop COVID-19 with mild symptoms, not requiring hospital admittance and thus escaping the laboratory confirmation of the disease [7]. Cancer patients undergoing treatment with anti-PD-1/PD-L1 or anti-CTLA-4 immune checkpoint inhibitors (ICI) currently used in everyday practice to treat solid tumors such as melanoma, lung cancer, renal carcinoma, urothelial cancers and head and neck carcinoma constitute a growing oncological population [8]. Their specific susceptibility to bacterial or viral infections has not been investigated. Considering that immunotherapy with ICI is able to restore the cellular immunocompetence, as we previously suggested in the context of influenza infection, the patient undergoing immune checkpoint blockade could be more immunocompetent than cancer patients undergoing chemotherapy [9,10].

## Potential interference between COVID-19 pathogenesis & immune checkpoint blockade

In the recent weeks, in the countries heavily interested by the COVID-19 outbreak, such as Italy, the scientific associations recommended the prudential postponing of active cancer treatments, especially for stable patients not needing urgent interventions [11]. On one hand, this recommendation could be reasonable for advanced cancer patients receiving chemotherapy, with the risk of hematological toxicity and of worsening an immunosuppressed status, thus favoring COVID-19 morbidity [5]. On the other hand, some oncologists are even currently wondering about the risk of administering ICI in the middle of the COVID-19 outbreak, essentially due to two major concerns.

The first seems to be represented by the potential overlap between the coronavirus-related interstitial pneumonia and the possible pneumological toxicity from anti-PD-1/PD-L1 agents. Even if lung toxicity is not the most frequent adverse event of ICI, it can be life threatening. The overall incidence rate of ICI-related pneumonitis ranges from 2.5–5% with anti-PD-1/PD-L1 monotherapy to 7–10% with anti-CTLA-4/anti-PD-1 combination therapy [12]. The dominant radiological pattern of lung immune-related adverse events (irAEs) is organizing pneumonia, but ICI-related pneumonitis could exhibit a variety of patterns, also including nonspecific interstitial pneumonitis [13]. Despite being rarer than other irAEs, pneumonitis is the most fatal AE associated with PD-1/PD-L1 inhibitor therapy, accounting for 35% of treatment-related toxic deaths [14]. Considering that underlying lung disease, particularly including interstitial pneumopathy, is considered a risk factor for ICI-related pneumonitis, it could be reasonable taking into account the risk of treating patients while they are developing an initial form of COVID-19. The synergy between the two lung injuries, despite only hypothetical, cannot be surely ruled out. Nevertheless, such an epidemiological coincidence should not prevent the oncologist from offering a potentially effective and often well-tolerated treatment even in the middle of the COVID-19 outbreak, since the duration of the pandemic is still currently unpredictable. This is true in particular considering the potentially curative aim of ICI treatment in the context of highly responsive diseases, such as melanoma and renal cell carcinoma and in the adjuvant setting even more than in the advanced disease.

The second concern seems to be represented by a possible negative interference of ICI in the pathogenesis of COVID-19. Cytokine-release syndrome (CRS) is a phenomenon of immune hyperactivation typically described in the setting of T cell-engaging immunotherapy, including CAR-T cell therapy but also anti-PD-1 agents [15]. CRS is characterized by elevated levels of IL-6, IFN- γ and other cytokines, provoking consequences and symptoms related to immune activation, ranging from fever, malaise and myalgias to severe organ toxicity, lung failure and death. In parallel, one of the most important mechanism underlying the deterioration of disease in COVID-19 is represented by the cytokine storm, leading to acute respiratory distress syndrome or even multiple organ failure [16]. The cytometric analyses of COVID-19 patients showed reduced counts of peripheral CD4 and CD8 T cells, while their status was hyperactivated. In addition, an increased concentration of highly proinflammatory CCR6+ Th17 in CD4 T cells has been reported, and CD8 T cells were found to harbor high concentrations of cytotoxic granules, suggesting that overactivation of T cells tends to contribute to the severe immune injury of the disease [17]. Moreover, the pathological findings associated with acute respiratory distress syndrome in COVID-19 showed abundant interstitial mononuclear inflammatory infiltrate in the lungs, dominated by lymphocytes, once again implying that the immune hyperactivation mechanisms are at least partially accountable for COVID-19 severity [17]. Considering these aspects, the hypothesis of a synergy between ICI mechanisms and COVID-19 pathogenesis, both contributing to a counter-producing immune hyperactivation, cannot be excluded.

In spite of this fascinating rationale, we should remember that ICI-induced CRS is a quite rare phenomenon as well as that the cytokine storm is not an early event in the COVID-19 pathogenesis, indeed characterizing the late phase of its most severe manifestation, occurring in a minority of patients. It is not likely that cancer patients are still receiving ICI during this phase of the viral illness. Obviously, in the current pandemic scenario, careful attention should be dedicated in delaying treatment for those patients presenting flu-like symptoms at the time of the intended ICI treatment.

## Therapeutic implications: tocilizumab & the risk of hasty conclusions

Since its first outbreak in China, COVID-19 was empirically treated with antiviral therapy, first employing agents already used in prior severe acute respiratory syndrome epidemics [18]. Then, several randomized clinical trials were initiated in China and more recently in Italy, investigating different treatment options, varying from classical antiviral drugs as lopinavir/ritonavir, to newer antiviral as remdesivir, to unconventional agents such as chloroquine and hydroxychloroquine [19]. The latest treatment frontier against COVID-19 seems to be represented by a recombinant humanized monoclonal antibody, named tocilizumab, which binds the human IL-6 receptor, inhibiting its signal transduction [20]. Tocilizumab is currently used for rheumatoid arthritis, but its efficacy has been demonstrated also against ICI-induced irAEs, starting from the rationale of an ICI-induced systemic inflammatory response syndrome similar to CRS [21]. Moreover, along with the improvement in symptoms related to systemic inflammatory response syndrome, some authors reported a clinical improvement in other irAEs with tocilizumab used in cancer patients with immune-related toxicity from anti-PD-1 agents [21,22].

With these premises, the risk of hasty conclusions is around the corner. In fact, one can argue that the alleged tocilizumab efficacy both for treating COVID-19 and irAEs might suggest a potentially increased danger from SARS-CoV-2 infection for ICI-treated patients, maybe hypothesizing a synergy in the promotion of the viral morbidity. Nevertheless, this is probably a thoughtless deduction.

First, it can be a matter of time. The time at which the COVID-19 patient develops the pathologic hyperactivation of the immune response, eventually contributing to the final injury, is probably in the late phase of the disease manifestation, occurring together with the respiratory distress [17]. Furthermore, the time matters also in the case of ICI therapy, since the majority of patients develop irAEs within the first 6 months from the first administration [12]. Thus, a certain caution for ICI administration during the pandemics could be applied mostly for those patients needing therapy initiation or in their first months of treatment.

Second, it is probably a matter of patient. Patients more prone to developing immune hyperactivation are probably those more likely to respond to ICI [23]. There is a possibility that such patients would be also more prone to fall in the cytokine storm in the case of SARS-CoV-2 infection. Nevertheless, these patients do not correspond to the average advanced cancer patient, who is supposed to be immunosuppressed, with a blunted immune status [6]. The epidemiology of the COVID-19 observed up today suggests that SARS-CoV-2 tends to infect more frequently the frail patient populations, such as the elderly and cancer patients [4,5]. Cancer is usually associated with overexpression of immunosuppressive cytokines, suppression of proinflammatory danger signals, impaired dendritic cell maturation, and enhanced immunosuppressive leukocyte populations [6]. Since ICI can restore the immune-competence, if on one hand it can be paradoxically needed to develop the cytokine storm characterizing the acute respiratory distress syndrome (ARDS) phase, on the other hand the epidemiological features of SARS-CoV-2 infection lay for a lower probability to affect these patients compared with their chemo-treated immune-suppressed counterpart.

Third, the efficacy of tocilizumab for COVID-19 is still under investigation, with still unexplored backstage and with uncomfortable upstream evidence coming from the setting of influenza infection. Despite clinical studies associating IL-6 with high disease severity in influenza-infected patients and its levels correlated directly with symptom occurrence in human influenza virus infection, the role of this cytokine is still ambiguous [24]. It was demonstrated in mice models that IL-6 is essential for preventing virus-induced neutrophil cell death and H1N1-associated mortality, limiting influenza-induced cytokine storm and protecting against fatal lung pathology [25]. Furthermore, IL-6 is crucial in secondary infections to recall virus-specific memory CD4 T cells, favoring virus clearance and host survival, as supported by the inability of IL-6 deficient mice to control influenza viral titers in the lung [25]. Such preclinical evidence suggests that, despite probably being harmful in the ARDS phase, IL-6 role could be crucial, in the early phase of the viral infection, to defuse the pathogenesis of severe and lethal forms of influenza. Thus, hoping for positive results from tocilizumab randomized clinical trial on COVID-19 patients, we could only argue about the evident diversity of this viral infection from previous SARS outbreaks and even more from influenza epidemics, probably both in terms of clinical features and of pathogenetic implications.

## Conclusion

Clinical decisions about cancer patients deserving immunotherapy in the current context of the COVID-19 pandemic should be characterized by separated reflections, avoiding generalizations and remembering their deeply different immunological status compared with that of cancer patients undergoing chemotherapy or targeted agents. In the end, beyond any charming scientific speculations, it is unfortunately likely that in this COVID-19 pandemic, the greatest risk for cancer patients is the unavailability of the usually high-level medical services, since all our hospital resources, in terms of structures, tools and healthcare professionals, are currently strongly dedicated to the outbreak management.
